# The reproductive microbiome and maternal transmission of microbiota via eggs in *Sceloporus virgatus*

**DOI:** 10.1093/femsec/fiae011

**Published:** 2024-02-02

**Authors:** Marie E Bunker, Stacey L Weiss

**Affiliations:** Department of Biology, University of Puget Sound, 1500 N. Warner Street, Tacoma, WA 98416, United States; Department of Biology, University of Puget Sound, 1500 N. Warner Street, Tacoma, WA 98416, United States

**Keywords:** egg microbiome, gut microbiome, high-throughput sequencing, maternal transmission, reproductive microbiome, reptiles

## Abstract

Maternal transmission of microbes occurs across the animal kingdom and is vital for offspring development and long-term health. The mechanisms of this transfer are most well-studied in humans and other mammals but are less well-understood in egg-laying animals, especially those with no parental care. Here, we investigate the transfer of maternal microbes in the oviparous phrynosomatid lizard, *Sceloporus virgatus*. We compared the microbiota of three maternal tissues—oviduct, cloaca, and intestine—to three offspring sample types: egg contents and eggshells on the day of oviposition, and hatchling intestinal tissue on the day of hatching. We found that maternal identity is an important factor in hatchling microbiome composition, indicating that maternal transmission is occurring. The maternal cloacal and oviductal communities contribute to offspring microbiota in all three sample types, with minimal microbes sourced from maternal intestines. This indicates that the maternal reproductive microbiome is more important for microbial inheritance than the gut microbiome, and the tissue-level variation of the adult *S. virgatus* microbiota must develop as the hatchling matures. Despite differences between adult and hatchling communities, offspring microbiota were primarily members of the *Enterobacteriaceae* and *Yersiniaceae* families (Phylum Proteobacteria), consistent with this and past studies of adult *S. virgatus* microbiomes.

## Teaser

Striped plateau lizard females vertically transmit microbes from their reproductive microbiome—specifically oviductal and cloacal microbes—to their offspring, via eggshell and egg contents.

## Introduction

Maternal transmission of microbiota has increasingly been documented across the animal world (Funkhouser and Bordenstein 2013, Baldo et al. 2018, Moeller et al. 2018, Youngblut et al. 2019), but the mechanisms of this transfer, and the heritability of the microbiome remains unclear and variable across taxa (Lauder et al. 2016, Youngblut et al. 2019). In mammals, bacteria are transferred during passage through the birth canal, and possibly during development in the womb, although that is currently under debate (Dominguez-Bello et al. 2010, Funkhouser and Bordenstein 2013, Stinson et al. 2019, Rowe et al. 2020, Kennedy et al. 2023). The neonatal microbiome is then supplemented through later parental care, which can vary widely across taxa, including nursing (Milani et al. 2017) and direct parent-to-offspring contact (Banning et al. 2008, Dominguez-Bello et al. 2010). The early inoculation of microbes is critical for healthy development of the adult microbiome, and can have long-term health consequences when perturbed (Lozupone et al. 2012, Knutie et al. 2017, Shao et al. 2019, Warne et al. 2019, Kim et al. 2020).

While less well-studied, there is also evidence that maternal microbes are transferred in oviparous animals (reviewed in Nyholm 2020). Oviparous animals show kinship effects in their microbiomes, with hatchlings from the same clutch harboring a more similar cohort of microbes than unrelated animals (Yuan et al. 2015, Trevelline et al. 2018, Ambrosini et al. 2019). For many species, this inoculation is dependent on behavioral mechanisms associated with parental care. For example, several bird species have been found to deposit skin, feather, preen oil, and fecal bacteria onto their eggs during incubation (Giraudeau et al. 2014, Martínez-García et al. 2015, van Veelen et al. 2018) and may influence the nest microbial environment through selection of plant materials with antimicrobial properties (Ruiz-Castellano et al. 2016). Some insect species will supply offspring with a specially produced capsule of obligate bacterial symbionts in the nesting environment (Hosokawa et al. 2006), and some species of squid have a specialized organ for depositing bacteria into the jelly coat of their eggs (Nyholm 2020).

For egg-laying animals that do not provide parental care (which includes over 99% of oviparous lizards and 97% of oviparous snakes; Richard 1987), inoculation with essential vertically transmitted microbes must occur during egg development or oviposition. Microbes found within egg contents and internal egg surfaces can be traced to the maternal gut or reproductive microbiome (Singh et al. 2014, van Veelen et al. 2018), indicating colonization during egg maturation. In chickens and rock pigeons, these microbes persist at least into the embryonic or hatchling stage (Lee et al. 2019, Dietz et al. 2020). Further, eggshells can be colonized with microbes from the cloaca during oviposition (van Veelen et al. 2018, Bunker et al. 2021, Li et al. 2022), and some bacterial species are able to penetrate eggshells (Gantois et al. 2009, Chen et al. 2019). These two potential pathways for maternal inheritance—through inoculation during egg development or penetration of the eggshell after oviposition—need further investigation, particularly as the maternal microbiome is not uniform throughout the digestive and reproductive tract (Kohl et al. 2017, Bunker et al. 2022), and different microbial cohorts may be passed down from different maternal tissues.

Here, we investigate pathways for maternal transmission of microbes in striped plateau lizards (*Sceloporus virgatus*), an oviparous phyrnosomatid lizard found in Mexico and parts of the southwestern USA. *Sceloporus virgatus* houses a specialized cloacal microbiome, which is known to be transferred to eggshell surfaces during oviposition, and to protect eggs from fungal infection during development (Bunker et al. 2021). Further, variation has been found in the microbiota of *S. virgatus* oviductal, cloacal, and intestinal tissue (Bunker et al. 2022), allowing for identification of the potential source of inherited microbes. These three maternal tissues are representative of the reproductive microbiome (oviduct), the gut microbiome (intestine), and the junction of the two (cloaca). This system offers the opportunity to determine if maternal transmission is occurring in the lizards, and if so, to assess potential pathways of this transmission.

## Methods

### Sample collection

A total of 10 gravid female striped plateau lizards [*S. virgatus*; mean snout-to-vent length (SVL): 66.7 ± 0.73 mm (SE)] were collected by lasso from one contiguous population following roughly 2 km of creekbed near the Southwestern Research Station in Cochise County, Arizona, on 26–27 June 2022. Gravidity was readily determined by palpation. Lizards were kept in 23 cm × 15 cm cages sterilized with 70% ethanol prior to animal capture and had sterile water available *ad libitum*, but were not fed. *S. virgatus* females do not oviposit in captivity without induction. Thus, on 29 June, after we observed females nesting in the field and we determined by palpation that eggs of captive females were fully turgid and ready for oviposition, we induced the captive females to oviposit via a 0.1-ml intraperitoneal injection of oxytocin (Andrews and Rose 1994). Each female oviposited in individual covered containers, lined with paper towel sanitized with 70% ethanol. Generally, oviposition began within ∼60 min of injection, and all females completed laying between 3.5 and 5.5 h of injection. We collected eggs as they were laid using sterile forceps, and weighed them (mean egg mass: 0.37 ± 0.002 g).

Clutch size ranged from 11 to 15 eggs. For each clutch, we destructively sampled the first and last laid egg for contents and eggshell (described below), we incubated two to three eggs in sterile vermiculite with no manipulation, and we treated the remaining eggs with a single species of fungal spores on Day 7 of incubation, as part of another study (see Table S1, Supporting Information). To acquire egg contents (*n* = 20), we punctured each egg with sterile scissors and extruded the entire contents (including the embryonic disk, yolk, and all other extraembryonic material) into sterile 1.5 ml microcentrifuge tubes. Emptied eggshells (*n* = 20) were stored in separate sterile microcentrifuge tubes. Efforts were made to keep eggshell and egg content samples distinct, but due to the nature of sampling there was still contact between eggshells and contents during collection. Incubated eggs (*n* = 105) were placed in individual containers filled with sterile vermiculite dampened with 0.8 ml sterile water per gram; containers were covered with parafilm and incubated at 30°C until hatching. A sample of sterile vermiculite, substrate from the laying surface, and a control swab of the researchers’ hands and general lab environment were taken as controls, to account for potential contamination.

On 30 June, females were euthanized with two injections of buffered tricaine methanesulfonate, according to Conroy et al. (2009), followed by decapitation. Tissue samples from the oviduct, cloaca, and intestine were taken with heat sterilized instruments. Oviduct and intestine were each sampled in two locations (oviduct: lower right and upper left oviduct; intestine: ∼2 mm above the cloaca and below the cecum), which were sequenced separately and treated as replicates, as the communities were similar. We took a single, internal cloacal tissue sample from directly above the vent. Substrate from the dissection surface was taken as a control, to account for potential contamination.

There was a 100% hatch success rate for incubated eggs. Within 24 h of hatching, hatchlings were removed from their incubation cups and immediately weighed (mean hatchling body mass: 0.42 ± 0.003 g), measured (mean SVL: 24.0 ± 0.06 mm), and sacrificed by decapitation. We sampled the entire intestinal tract, from just below the stomach to the cloaca, using a dissecting microscope and heat sterilized instruments, and wearing gloves sterilized with 70% ethanol. We did not subsample the intestine due to its small size (mean mass of hatchling tissue samples: 3.6 mg ± 0.19). Substrate from the dissection surface, which was sterilized with 70% ethanol between each hatchling, was taken as a control, to account for potential contamination.

All samples were stored at −80°C until extraction. All methods were approved by the University of Puget Sound Institutional Animal Care and Use Committee (IACUC #PS21003). Fieldwork was conducted under Arizona Game and Fish Department License SP762402 and US Forest Service Special Use Permit DOU2223.

### DNA extraction and Illumina library prep

We extracted DNA from all tissue samples using the Qiagen DNEasy Blood and Tissue Kit (Qiagen, Inc), including the optional lysis buffer incubation for 30 min at 37°C. Samples were incubated at 56°C for 180 min while shaking at 500 RPM. Eggshells were rinsed with 200 µl sterile phosphate buffered saline (PBS) prior to extraction to remove any remaining vermiculite. We extracted DNA from eggshells similarly to tissue samples, with the addition of a bead beating step in which two sterile 2.8 mm ceramic beads were added to tubes prior to the second incubation and samples were vortexed for 15 min at top speed. Eggshells were incubated at 56°C for 90 min while shaking at 500 RPM. We extracted egg contents using the Qiagen PowerSoil Kit, following manufacturer protocols. All extractions included an extraction blank as negative control, and PBS was added to the blank for relevant extractions.

Briefly, we prepared Illumina libraries via a two-step polymerase chain reaction (PCR), in which PCR1 amplified the V4 region of the 16 s rRNA gene via 515F/806R primer pairs, and PCR2 added unique barcodes to each sample. Samples were pooled with varying volumes to qualitatively match DNA concentration, based on PCR band strength, and sent to the University of Idaho Genomics and Bioinformatics Core for clean up and sequencing on the Illumina MiSeq platform (v3, 2 × 300). More details can be found in Bunker et al. (2021, 2022). A mock community was also amplified and included in sequencing as a positive control and to inform data processing.

### Raw data processing

We received sequences demultiplexed, with adapters and primers removed. All raw data files have been uploaded to the NCBI Sequence Read Archive and can be accessed here: http://www.ncbi.nlm.nih.gov/bioproject/1023543. Inspection of the mock community and quality scores generated with FastQC (Andrews 2010) and aggregated with MultiQC (Ewels et al. 2016) were used to determine all parameters used in preparation of raw data. Samples were processed in R v 4.1.2 using the DADA2 pipeline (Callahan et al. 2016a,b). Samples were trimmed at 260 base pairs for forward reads and 190 for reverse, and filtered with a maximum expected error of 2. Taxonomic classification of amplified sequence variants (ASVs) was performed through the assignTaxonomy function, using the Silva database (Quast et al. 2013), release 138. Potential contaminants were removed with the Decontam package (Davis et al. 2018), using the “prevalence” method with a threshold of 0.1. Control samples (*n* = 31) included environmental controls from field collection, laying environment, and dissection surfaces, experimental controls, extraction blanks, and PCR negatives. We discarded any ASV that had fewer than 27 reads across all samples (based on analysis of mock communities) and all nontarget (nonbacterial) ASVs. Processed reads were used to generate and optimize phylogenetic trees using the DECIPHER and Phangorn packages (Wright 2016, Schliep et al. 2017). Finally, read numbers were log transformed to account for differences in read depth, as rarefaction curves indicate sufficient diversity was captured for all samples, and thus rarefaction was not necessary (Figure S1, Supporting Information). We excluded samples which had fewer than 500 reads after processing from the analysis (*n* = 9). Final sample sizes are in Table 1. All figures were made using the ggplot2 package (Wickham 2017).

**Table 1.** Final sample size used in analyses for each sample type.

Sample type	*N*
Egg contents	18
Eggshells	19
Total hatchling tissue	99
Control* hatchling tissue	23
Maternal oviduct	18
Maternal cloaca	10
Maternal intestine	20

* Eggs not treated with fungus on Day 7 of incubation.

### Statistical analysis

All analyses were performed in R (version 4.1.2) (R Core Team 2020). Phyloseq (McMurdie and Holmes 2013 et al. 2013) was used to organize data of different types (i.e. ASV counts, taxonomy, and metadata). Shannon diversity and richness values were generated using the “estimate_richness” function from phyloseq, and Faith’s phylogenetic diversity (PD) was estimated using the described phylogenetic trees and the Picante package (Kembel et al. 2010). Principal coordinates analysis (PCoA) plots were generated using the “ordinate” function, and weighted and unweighted UniFrac distance matrices were generated using the function “UniFrac,” both from the phyloseq package.

To investigate the potential for maternal microbiota transmission, we assessed the impact of maternal identification on hatchling intestinal microbiota. All successfully hatched offspring were included in these analyses as the fungal treatment did not have an effect on offspring microbiota (Figures S2 and S3, Supporting Information), and treatments were equally represented within a clutch (Table S1, Supporting Information). Effect of maternal ID on alpha diversity was tested with ANOVAs, controlling for extracted hatchling tissue mass in milligrams, after determining that the models met assumptions of normality and dispersion. Only richness was log transformed to meet these assumptions. Overall effect of clutch on community membership and composition (also controlling for sample mass) was tested with permutational ANOVAs (permANOVA) using the “adonis2” function from the vegan package (Oksanen et al. 2019). Community dispersion was tested with the “betadisper” function, also from vegan.

To examine pathways of transmission from maternal tissues (oviduct, cloaca, and intestine) to all three offspring sample types (egg contents, eggshells, and intestine), the remaining analyses only included intestinal samples from “control” hatchlings, which were incubated in a sterile environment with no fungal treatment (*n* = 2–3 per clutch; Table S1, Supporting Information). First, we compared alpha and beta diversity metrics across all maternal and offspring sample types. Alpha diversity was compared using ANOVAs with maternal ID as a block, and again only richness was log transformed to meet assumptions. Tukey HSD tests were used for *post hoc* pairwise comparisons. Overall community composition and structure were compared with permANOVAs and beta dispersion, as described above. *Post hoc* pairwise comparisons were performed with the pairwiseAdonis package (Martinez 2017). Second, we used SourceTracker (Knights et al. 2011) to identify the likely maternal source tissue for offspring microbes, with all maternal tissues defined as “source” and all offspring sample types as “sink.” SourceTracker identifies the likely source of ASV’s but does not assess community structure. Thus, third, we compared community composition of individual offspring sample types to all maternal tissues. PCoA plots were generated using weighted and unweighted UniFrac distance matrices. Each plot was grouped into clusters of samples with k-means clustering. The number of clusters for each plot was determined based on elbow plots and average silhouette width values. Clusters were generated with the “pam” function from the cluster package (Maechler et al. 2022).

Finally, to assess variation in microbe provisioning within a clutch, we compared the egg content and eggshell microbiota of the first and last egg laid by each female. The influence of lay order on alpha diversity was examined using paired *t*-tests; all metrics except eggshell Shannon diversity were log transformed to meet test assumptions. The influence of lay order on beta diversity was examined using beta dispersion tests and permANOVAs, controlling for maternal ID, as described above.

Data analysis files have been submitted to the Dryad data repository, and can be accessed here: https://datadryad.org/stash/share/MGM1O8QrDcWxRdjIoUaudfk-e_GTK0G_GPHQhTsmzpA

## Results

### Offspring sample community compositions

**Figure 1.**
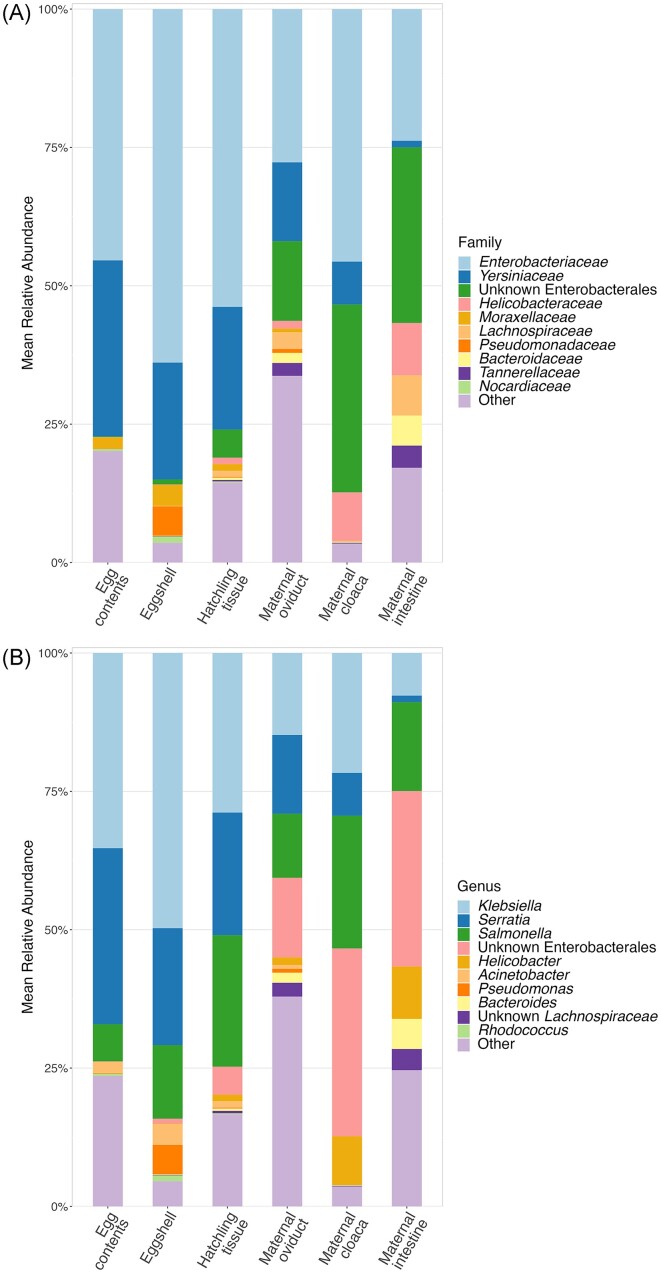
Average communities of all sample types. Mean relative abundances of top 10 most abundant Families (A), and Genera (B) across sample types, with the remaining taxa grouped into the “Other” category. Colors represent different taxa, and the height of each bar represents the average % of total reads assigned to each taxa, in each sample type.

For all three offspring sample types, the most abundant families were *Enterobacteriaceae* and *Yersinaceae* (Phylum Proteobacteria; Figure S4, Supporting Information). Members of the *Enterobacteriaceae* family made up 45.4 ± 7.1% of the egg contents community, 63.8 ± 7.6% of the eggshell community, and 53.8 ± 3.4% of the hatchling intestine community, on average (Fig. 1A). Members of the *Yersinaceae* family made up 31.8 ± 7.4% of the egg contents community, 21.1 ± 7.2% of the eggshell community, and 22.2 ± 3.2% of the hatchling intestine community, on average. The most common and abundant member of *Enterobacteriaceae* was *Klebsiella*, while all *Yersinaceae* ASVs were *Serratia* or unidentified at the genus level (Fig. 1B). No other family made up more than 5% of the average egg contents community. Eggshells and hatchling tissues each had one additional family accounting for ∼5% of the community on average (*Pseudomonadaceaea* and an unknown Enterobacterales, respectively), with the remainder of the community for all three sample types made up of low abundance taxa. The composition of maternal tissues have been described elsewhere (Bunker et al. 2022) and are generally similar here with a dominance of Proteobacteria families *Enterobacteriaceae* and *Yersinaceae* (previously classified as *Enterobacteriaceae*; Janda and Abbott 2021).

### Maternal transmission

We used intestinal tissues from all hatched offspring tissue to identify effects of maternal ID on hatchling microbiota. We found that all three alpha diversity measures of hatchling intestine tissue (Shannon diversity index, richness, and PD) varied depending on maternal ID (F_9,89_ ≥ 3.04, *P* ≤ .018; Table S2, Supporting Information). As well, both beta diversity metrics of hatchling intestine tissue varied with maternal ID (weighted and unweighted UniFrac; *P* = .001 for both; Table S3 and Figure S5, Supporting Information), with maternal ID accounting for 21% of variation in the hatchling microbiota based on weighted UniFrac distance, and 16% based on unweighted UniFrac distance. Samples were also dispersed differently between clutches based on unweighted UniFrac distance (F_9,89_ = 2.51, *P* = .013, Table S3, Supporting Information), although not weighted UniFrac (F_9,89_ = 1.37, *P* = .212, Table S3, Supporting Information).

### Pathways of transmission

**Figure 2.**
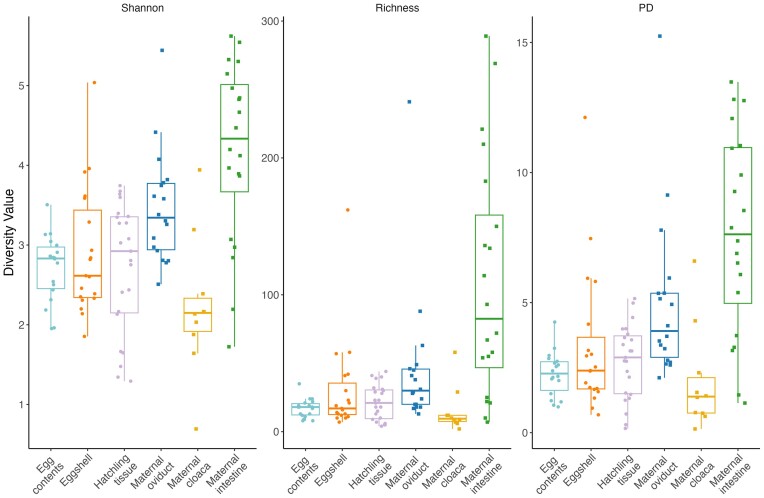
Shannon Diversity index values, Richness, and Faith’s PD index values for all sample types. Points represent individual diversity value for each sample, boxes represent median and quartiles of group diversity. The shape of the points indicates maternal (squares) or offspring (circles) samples.

**Figure 3.**
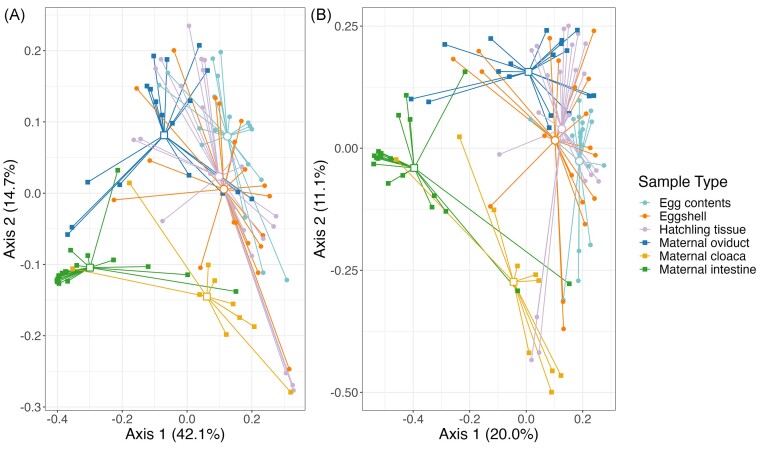
PCoA plots based on weighted (A) and unweighted (B) UniFrac distances, comparing three offspring sample types and three maternal tissue sample types. Colors of points and lines represent different sample types, and the shape of points indicates maternal (squares) or offspring (circles) samples. Filled points represent individual samples, and larger open points represent centroids from each group.

To examine potential pathways of microbe transmission, we compared egg contents, eggshells, and control hatchling intestine tissue (i.e. animals hatched from eggs which did not receive a fungal inoculation) to three maternal tissue types: oviduct, cloaca, and intestine. ANOVAs of all alpha diversity measures indicate an overall difference in diversity between all maternal and offspring sample types (F_5,93_ ≥ 2.31, *P* ≤ .022; Table S4, Supporting Information; Fig. 2). Based on *post hoc* tests, alpha diversity of egg contents, eggshells, and hatchling tissues were similar to the maternal cloaca and lower than the maternal intestinal tissue, in all metrics (Table S5, Supporting Information). Relative to the maternal oviductal tissue, egg contents had significantly lower diversity in all measures, eggshells had statistically similar diversity in all measures, and hatchling tissues had significantly lower diversity for Shannon and PD, but not for richness (Table S5, Supporting Information). PermANOVAs and pairwise permANOVAs indicate differences between the community structure of all sample types (*P* ≤ .010; Tables S6 and S7, Supporting Information), although patterns of overlap were still observed between groups on PCoA plots (Fig. 3), which were explored further with clustering analyses (see below).

**Figure 4.**
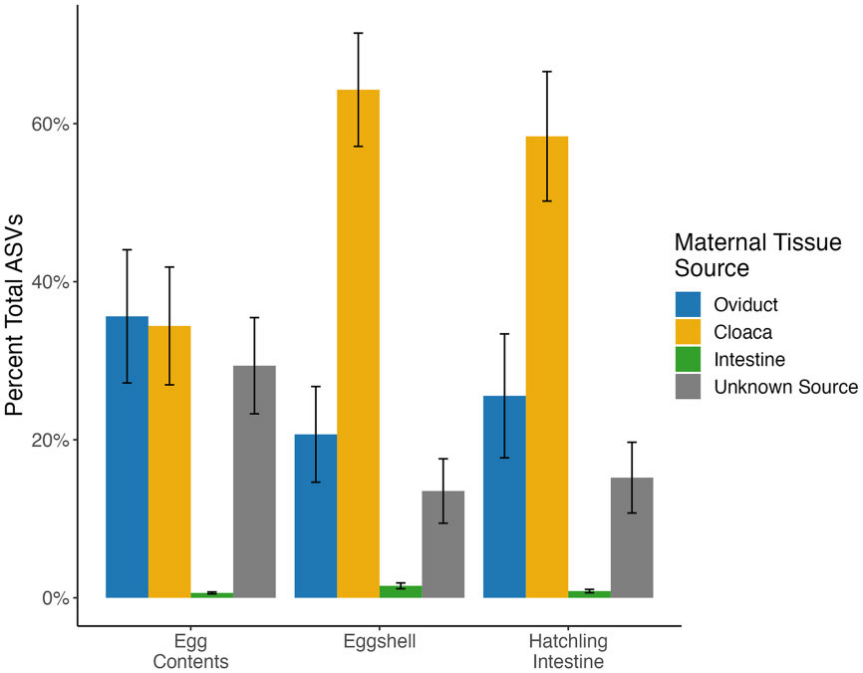
Average % of ASVs sourced from each maternal tissue type or unknown source (represented by colored bars) for each offspring sample type.

The SourceTracker analysis found that an average of 35.6% of ASVs in the egg contents were sourced from the maternal oviduct and 34.4% of ASVs were sourced from the maternal cloaca (Fig. 4, Supplemental File 2). In contrast, the majority of ASVs found on eggshells and in hatchling tissue were sourced from the maternal cloaca on average (58.4% and 64.3%, respectively), with most of the remaining ASVs coming from the oviduct (25.6% of ASVs on eggshells and 20.7% of ASVs in hatchling tissue) (Fig. 4, Supplemental File 2). For all offspring tissue types, a relatively low percentage of ASVs were sourced from the maternal intestine: 0.6% in egg contents, 1.0% on eggshells, and 1.5% in hatchling tissue (Fig. 4, Supplemental File 2). The source of the remaining taxa was unidentified.

**Figure 5.**
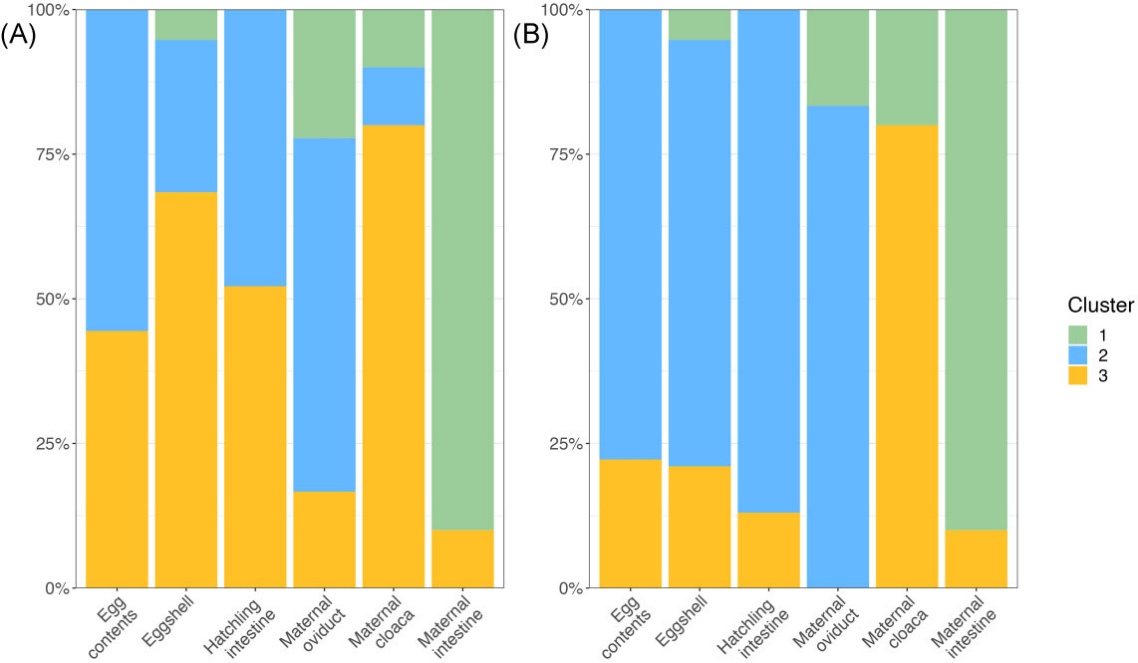
The % of each sample type assigned to each cluster (most closely grouped samples based on cluster analysis) for weighted UniFrac distance (A), and unweighted UniFrac distance (B). Colors represent each cluster, and height of each color represents the % of each sample type assigned to that group.

While the SourceTracker analysis identifies the likely maternal source of offspring ASVs, it does not assess the community structure of each offspring sample type relative to maternal sample types. Thus, we used cluster analysis based on the PCoA plots to assess whether the composition of offspring samples tend to cluster with a given maternal sample type. Using k-means clustering, samples were grouped into three clusters, with each cluster primarily associated with one of the maternal tissue types (Fig. 5). When using weighted UniFrac distance, Cluster 1 contained 90% of the maternal intestine tissues, only 22% of maternal oviductal and 10% of maternal cloacal tissue samples, and only a single eggshell sample, with no egg contents or hatchling tissues. Cluster 2 included 61% of the oviductal samples and 10% of the cloacal samples, but none of the maternal intestinal samples. It also included the majority of the egg contents (56%), 48% of hatchling intestine samples, and 26% of eggshells. Cluster 3 contained 80% of the maternal cloacal samples, 16% of the maternal oviduct samples, and the remaining 10% of maternal intestine samples, as well as 44% of egg contents, 52% of hatchling intestines, and 68% of eggshells (Fig. 5A). Using unweighted UniFrac, again, Cluster 1 contained most of the maternal intestine samples (90%), Cluster 2 contained most of the maternal oviduct samples (83%), and Cluster 3 contained most of the maternal cloacal samples (80%). All three offspring sample types predominantly fit into Cluster 2: 78% of egg contents, 73% of eggshells, and 87% of hatchling intestine, with nearly all other samples fitting into Cluster 3 and a single eggshell sample fitting into Cluster 1 (Fig. 5B).

### Within-clutch variation in transmission

**Figure 6.**
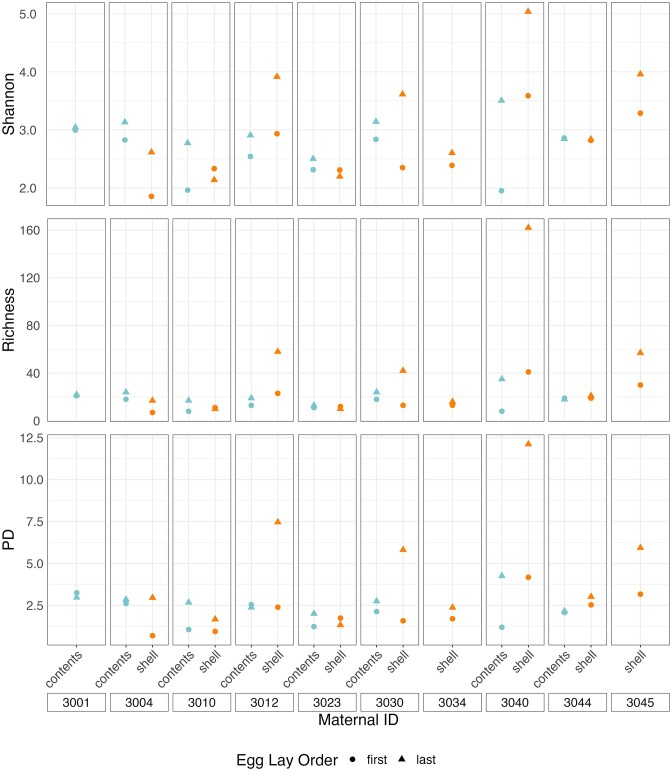
Shannon Diversity index values, Richness, and Faith’s PD index values for egg contents and eggshells of first and last laid egg in each clutch. Colors represent sample types and shapes represent egg lay order.

**Figure 7.**
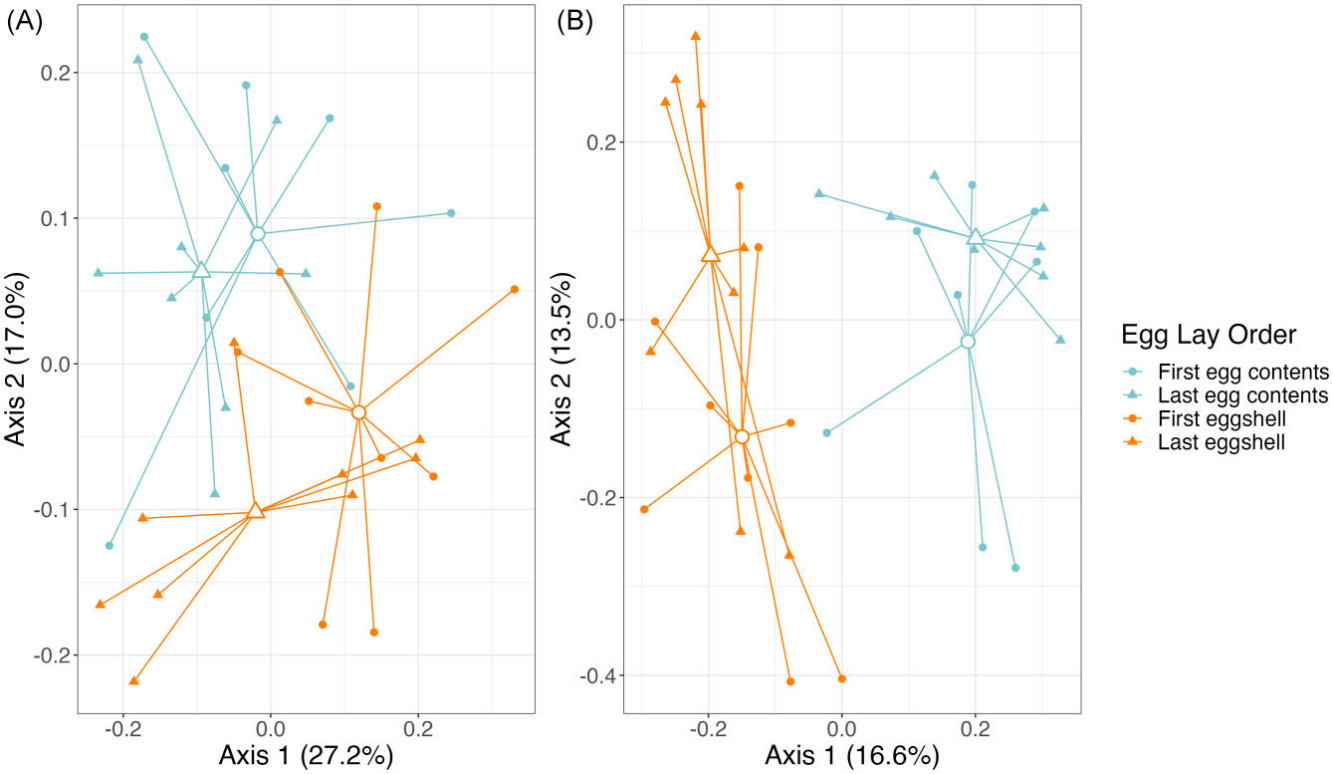
PCoA plots based on weighted (A) and unweighted (B) UniFrac distances, comparing egg contents and eggshells of first and last laid egg in each clutch. Colors represent sample types and shapes represent egg lay order. Closed shapes represent individual samples, and larger open shapes represent centroids from each group.

We found that contents of the first egg had significantly lower alpha diversity than did that of the last egg for both Shannon Diversity and Richness (*t*_7_ ≤ −2.41, *P* ≤ .047, Fig. 6), and there was a similar trend for PD (*t*_7_ = −2.08, *P* = .077; Table S8, Supporting Information; Fig. 6). There was no variation in community structure or membership (Table S9, Supporting Information; Fig. 7A and B). The shell of the first egg laid in each clutch had significantly lower diversity than the shell of the final egg in all three alpha diversity measures (*t*_8_ ≤ −2.78, *P* ≤ .024; Table S8, Supporting Information; Fig. 6). Overall community structure (weighted UniFrac, *P* = .011, Fig. 7A) and membership (unweighted UniFrac, *P* = .043, Fig. 7B) differed between the first and last eggshells, but dispersion did not (Table S9, Supporting Information).

## Discussion

We found that maternal ID impacted the microbiota of hatchling tissue, which supports the hypothesis that maternal transmission is occurring in *S. virgatus*. The microbes are transferred primarily from the cloaca and oviduct during egg development and oviposition, with very little impact from intestinal microbes. Both egg contents immediately after oviposition and offspring tissues immediately after hatching most closely resemble the oviductal community. This indicates that microbes may colonize the egg during development, possibly before the shell is developed, and these microbes persist through development into the hatchling intestine. This has been observed in chickens, in which the early egg white microbiome as well as the embryonic gut most closely resembled the oviductal community (Lee et al. 2019). Similarly, the gut microbiota of Amazon river turtle hatchlings is dependent on the early egg microbiota, although it was largely acquired from environmental microbes rather than maternal tissues (Carranco et al. 2022).

There is also a significant influence of cloacal microbes, which make up the majority of the taxa in all offspring communities, despite the overall structure of the communities being most similar to the oviduct. These cloacal microbes appear to be primarily transferred via the outer eggshell. This shell microbiome is known to protect eggs from fungal fouling during incubation and has also been associated with increased hatchling fitness (Bunker et al. 2021). The major taxa in both the maternal cloaca and eggshells belong to the *Yersinaceae* and *Enterobacteriaceae* families, which have been identified as important functional taxa in adult cloacal microbiomes, as they provide antifungal protection to eggs during development in the soil (Kalbe et al. 1996, Dhar Purkayastha et al. 2018, Bunker et al. 2021). This indicates some selection for these microbes, and could explain the preferential vertical transmission observed here. Some bacterial taxa are able to penetrate eggshells (Gantois et al. 2009), so it is possible that these microbes are deposited on the shell only during oviposition and establish in the embryo during incubation. It has also been hypothesized that microbes can ascend to the reproductive tract from the vagina (in humans; Hansen et al. 2014) or gut (in chickens; Shterzer et al. 2020), raising the possibility that the “cloacal” microbes also establish in the internal egg environment prior to shelling in the oviduct.

Because offspring communities have a high percentage of cloacal taxa but relatively low similarity with the cloacal community structure, there must be physiological or environmental properties, which favor the growth of certain taxa in eggs and hatchlings as they develop. For example, egg albumen is known to have antimicrobial properties (Shawkey et al. 2008), which may select for bacteria which are resistant to the antimicrobial peptides present in the egg. Further, the cloaca may be a more aerobic region than the upper areas of the oviduct and gut, which could impact how well the associated microbes persist in the egg environment during incubation and in the different regions of the hatchling gut or reproductive tracts (Shawkey et al. 2008, Videvall et al. 2018, Berlow et al. 2020). This could also account for the overall variation between sample types, as only a subset of the maternal bacteria survived in the egg environment.

The variation between first eggshell and last eggshell communities also indicates that microbes are not evenly provisioned to offspring. Given that the same patterns were not observed in the egg contents, variation in eggshell microbiota likely occurred after the eggshell formed. Eggs that develop higher in the oviduct, and thus have to pass through the entire length of the oviduct during oviposition would likely be exposed to a greater diversity of microbes, impacting the eggshell microbiota but not the egg contents at the time of sampling. van Veelen et al. (2018) found that the eggshell microbiome of first and second eggs in two lark species varied in the relative abundance of particular taxa, but these microbes were sourced to the nest environment rather than the maternal tissues. More research across oviparous taxa is needed to assess the effect of lay order on eggshell microbiota and the sources of those microbes.

Unlike the cloacal and oviductal microbes, we found little evidence that maternal intestinal microbes colonize eggs or hatchlings. Studies in chickens have found that gut microbes are transmitted to eggshells (Ding et al. 2017, Shterzer et al. 2020), although in *S. virgatu*s the distinct differentiation of the cloacal microbiome and inability of fecal microbes to establish at the cloaca (Bunker et al. 2022) make it less likely that eggs would come into direct contact with upper gut microbes. However, the lack of intestinal microbes was surprising in the hatchling tissue samples as the entire gut region was sampled. This indicates that variation between gut and reproductive tissues develops as the animals age, and that the adult intestinal microbes are likely acquired from diet or the environment. There is evidence in humans (Koenig et al. 2010), other mammals (Wang et al. 2019), birds (Hird et al. 2014, Taylor et al. 2019, Videvall et al. 2019, Hernandez et al. 2021), and other reptiles (Yuan et al. 2015) that the microbiome undergoes large structural changes both immediately after birth/hatching and as animals age from juveniles to adults, which may also account for differentiation of gut regions. This change could also be instigated when the animals begin foraging, offering an interesting area of investigation for future research.

This study was conducted in a controlled, sterilized environment, and does not account for the potential impact of nest microbes, which are known to be integral to development of the neonate microbiome in other species (van Veelen et al. 2018, Campos-Cerda and Bohannan 2020). Although the lack of parental care means that *S. virgatus* nests are not manipulated in the way that bird and other reptile nests are, the nest microbiome has still been shown to influence offspring microbes in other reptiles that also lack parental care (Carranco et al. 2022, Li et al. 2022). We cannot rule out the possibility that soil microbes penetrate the shell during incubation to colonize the embryo, leading to a different hatchling microbiota in wild hatchlings than the one recovered in this study. Additionally, we noted the presence of the incubation medium (vermiculite) in the hatchling intestines upon dissection, indicating the possibility that hatchlings ingest soil as they leave the nest. This could serve as an initial inoculation with soil microbes, altering the wild hatchling microbiota and accounting for microbial variation found in other lizard species based on habitat or geographic location (Baldo et al. 2018, Alemany et al. 2022, Bunker and Weiss 2022).

Finally, there are a large percentage of microbes present in hatchlings that were not found in any of the maternal tissues we sampled, and it is unclear where those taxa are originating from. It is possible that the taxa are present in low levels in the maternal tissues, but were only detectable in the relatively lower bioload environment of the offspring samples. Some of these taxa may originate from tissues which were not sampled in this study, particularly the ovaries, which have been hypothesized as a source for gecko egg microbes (Singh et al. 2014) or the upper intestine, which is known to be differentiated from the lower intestine and oviduct in *S. virgatus* (Bunker et al. 2022). Another possibility is that these microbes could originate from the paternal ejaculate microbiome; while less well studied than the female reproductive microbiome, microbes have been isolated from sperm and in some cases can impact reproductive success (Rowe et al. 2020).

Overall, these results establish that maternal transmission of microbes is occurring in *S. virgatus*, and much of that transfer occurs prior to oviposition, within the oviduct. More research must be done to see how this transfer is complicated by soil microbes, how the microbiome differentiates in different tissues over time, and the impact of paternal microbes. These findings can serve as a baseline to identify potential pathways of maternal transmission in other oviparous animals, and shift the primary focus from the gut to the reproductive microbiome.

## Supplementary Material

fiae011_Supplemental_Files
