# Supplementary figures and images for: The reproductive microbiome and maternal transmission of microbiota via eggs in Sceloporus virgatus
Source: FEMS Microbiol Ecol. 2024 Feb 2;100(3):fiae011. doi: 10.1093/femsec/fiae011 (PMC10873522; doi:10.1093/femsec/fiae011)

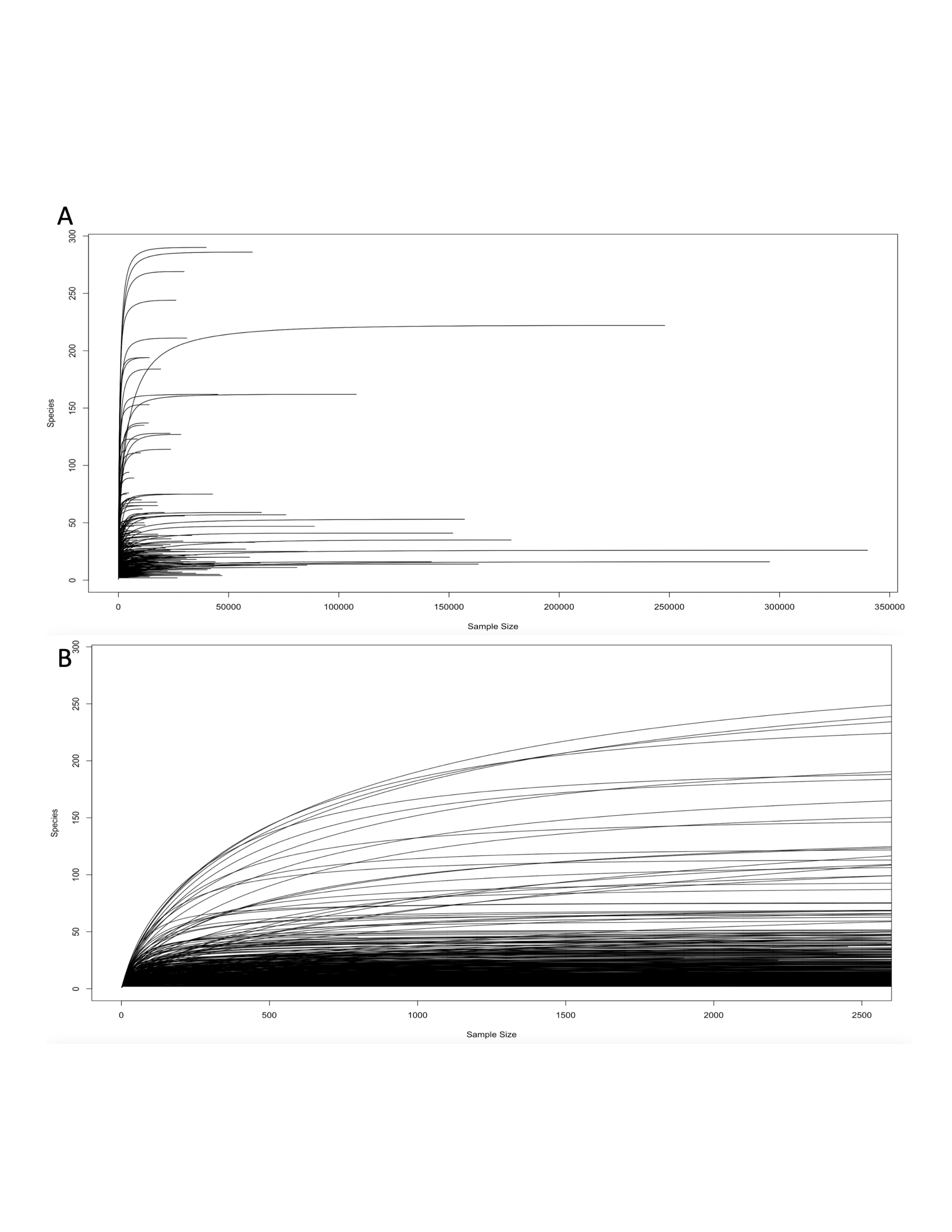

Supplement: fiae011_Supplemental_Files [file fiae011_supplemental_files.zip › Supplementary_data FigS1.png]

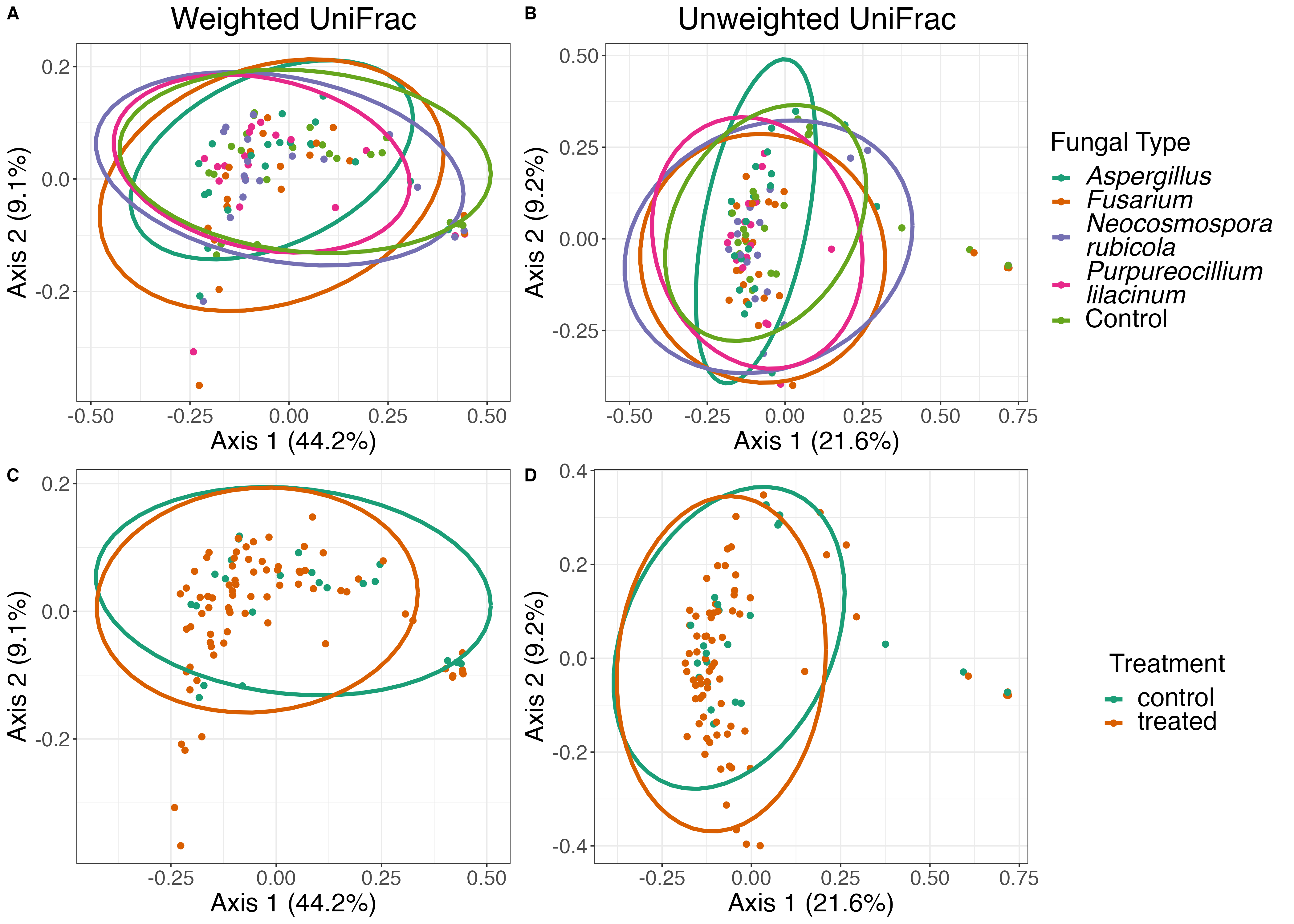

Supplement: fiae011_Supplemental_Files [file fiae011_supplemental_files.zip › Supplementary_data FigS2.png]

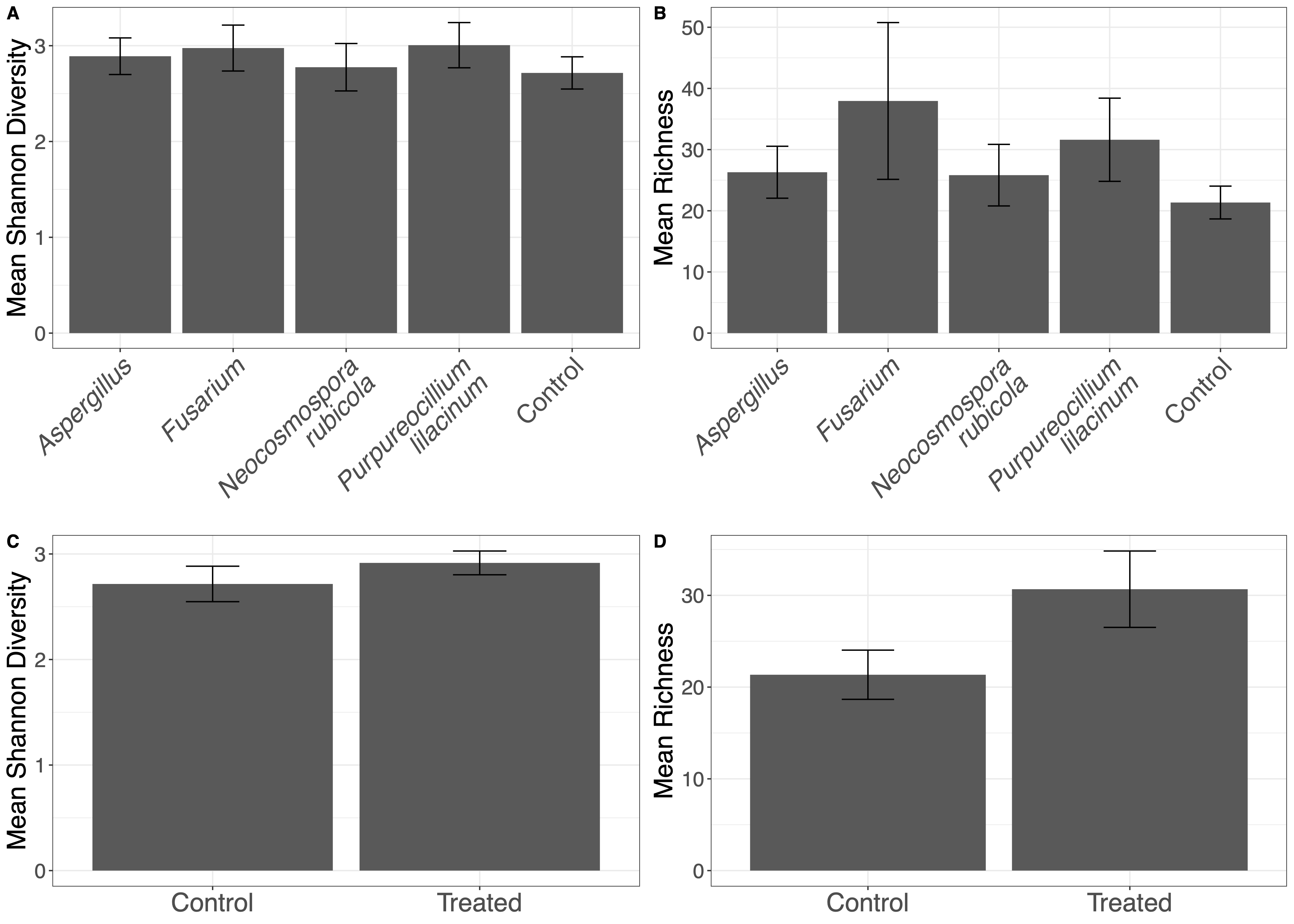

Supplement: fiae011_Supplemental_Files [file fiae011_supplemental_files.zip › Supplementary_data FigS3.png]

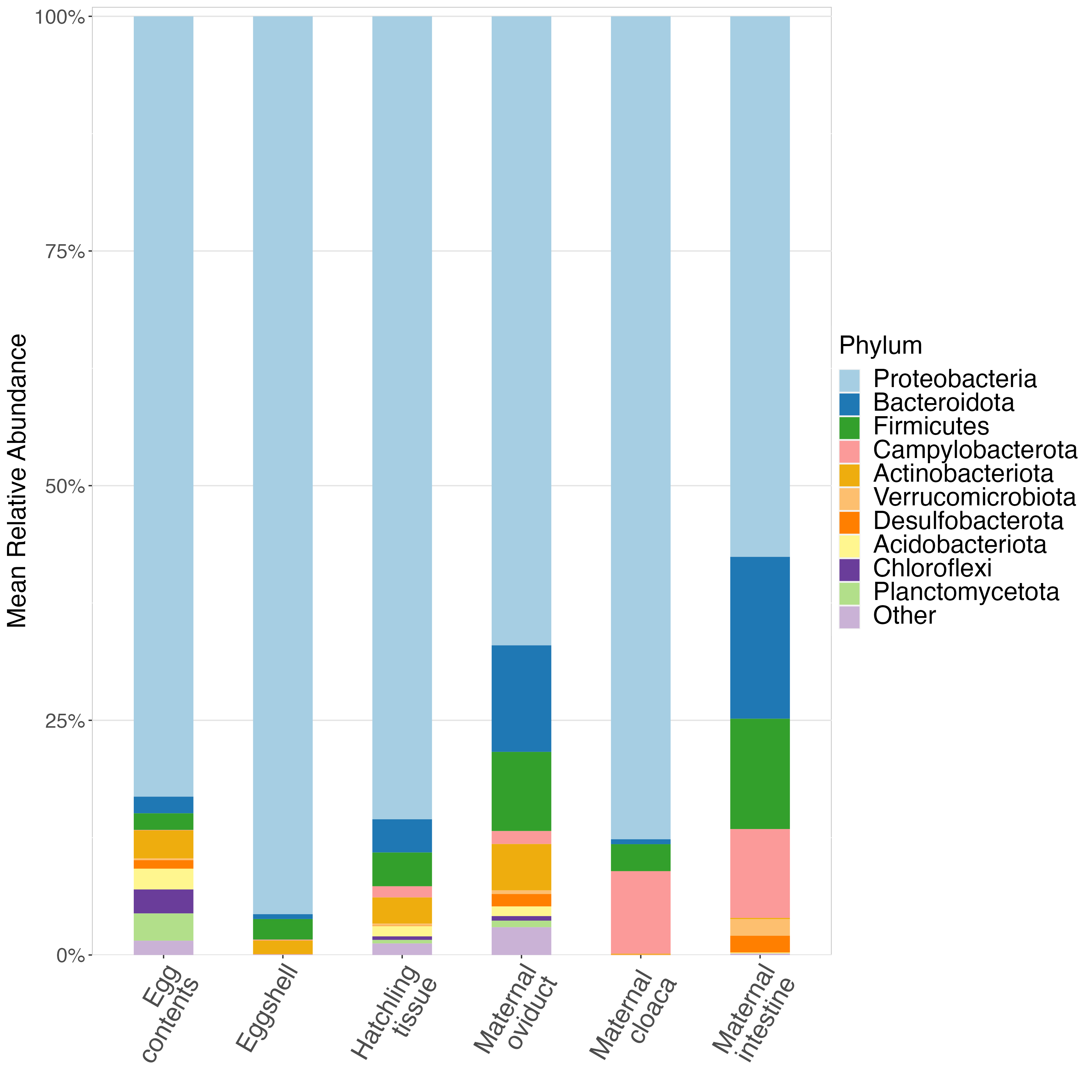

Supplement: fiae011_Supplemental_Files [file fiae011_supplemental_files.zip › Supplementary_data FigS4.png]

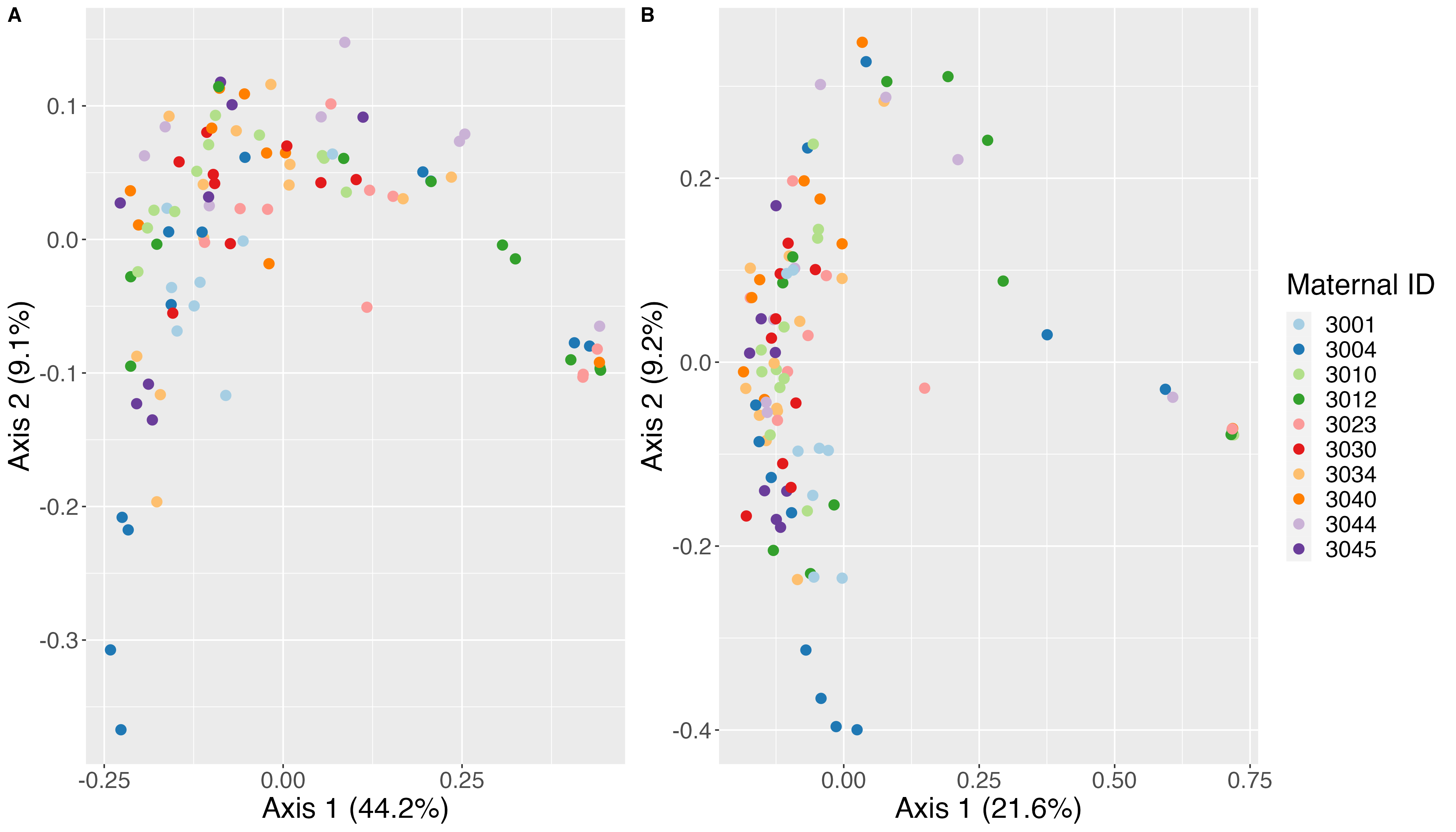

Supplement: fiae011_Supplemental_Files [file fiae011_supplemental_files.zip › Supplementary_data FigS5.png]
